# Allograft or Recipient ST2 Deficiency Oppositely Affected Cardiac Allograft Vasculopathy *via* Differentially Altering Immune Cells Infiltration

**DOI:** 10.3389/fimmu.2021.657803

**Published:** 2021-03-18

**Authors:** Zhenggang Zhang, Na Zhang, Junyu Shi, Chan Dai, Suo Wu, Mengya Jiao, Xuhuan Tang, Yunfei Liu, Xiaoxiao Li, Yong Xu, Zheng Tan, Feili Gong, Fang Zheng

**Affiliations:** ^1^ Department of Immunology, School of Basic Medicine, Tongji Medical College, Huazhong University of Science and Technology, Wuhan, China; ^2^ Key Laboratory of Organ Transplantation, Ministry of Education, NHC Key Laboratory of Organ Transplantation, Key Laboratory of Organ Transplantation, Chinese Academy of Medical Sciences, Wuhan, China

**Keywords:** IL-33, ST2, heart transplantation, cardiac allograft vasculopathy, chronic rejection

## Abstract

The role of IL-33/ST2 signaling in cardiac allograft vasculopathy (CAV) is not fully addressed. Here, we investigated the role of IL-33/ST2 signaling in allograft or recipient in CAV respectively using MHC-mismatch murine chronic cardiac allograft rejection model. We found that recipients ST2 deficiency significantly exacerbated allograft vascular occlusion and fibrosis, accompanied by increased F4/80^+^ macrophages and CD3^+^ T cells infiltration in allografts. In contrast, allografts ST2 deficiency resulted in decreased infiltration of F4/80^+^ macrophages, CD3^+^ T cells and CD20^+^ B cells and thus alleviated vascular occlusion and fibrosis of allografts. These findings indicated that allografts or recipients ST2 deficiency oppositely affected cardiac allograft vasculopathy/fibrosis *via* differentially altering immune cells infiltration, which suggest that interrupting IL-33/ST2 signaling locally or systematically after heart transplantation leads different outcome.

## Introduction

Despite improvement in short-term patient survival after heart transplantation (HTx), long-term survival rates have not improved much, mainly because of Cardiac Allograft Vasculopathy (CAV) (1). CAV is a major cause of graft failure in cardiac transplant recipients who survives more than 1 year after transplantation, with the morbidity of CAV exceeding 50% at 5 years (2). CAV is primarily characterized as an immune-mediated pan-arterial disease with intimal hyperplasia and narrowing of the allograft artery (3). Recent insights have underscored the fact that innate and specific immune responses are involved in the pathogenesis of CAV (4, 5). Because individual responses to an allograft change over time, assays to monitor the recipient’s immune response and individualized methods for therapeutic immune modulation are clearly needed. Nevertheless, past extensive studies, the molecular mechanisms underlying cardiac allograft vasculopathy are not yet to be fully elucidated (6). Up to now, there have been no enough strategies to effectively prevent CAV (7). IL-33 is a member of the IL-1 family (8). IL-33 has been identified to be the special ligand for the unique receptor ST2 (9). The IL-33/ST2 signaling has emerged as a pathway with a central role in processes of the immune response and homeostasis (10). At present, some studies have demonstrated that IL-33 as a novel alarmin in heart transplantation that limits CAV *via* restraining the local activation of macrophages (11, 12). IL-33 expression has been reported in the coronary artery (8, 13). How IL-33/ST2 signaling pathway is modulated in CAV and the functional effects of IL-33/ST2 is remained unknown. Based on these findings, we examined the role and functional significance of IL-33/ST2 signaling in cardiac allogeneic transplantation in mice. Our data indicated that grafts or recipients IL-33/ST2 signaling oppositely affected CAV *via* differentially altering immune cells infiltration.

## Materials and Methods

### Animals

C57BL/6 (B6, H-2^b^) mice, males, aged 7-9 weeks, were purchased from Shanghai SLAC Laboratory Animal Center, Chinese Academy of Sciences (Shanghai, China). B6.C-H-2bm12KhEg (bm12, H-2^bm12^) males, strains of mice B6 arose through a spontaneous mutation in the MHC-II I-A^b^ molecule (14), were purchased from the Jackson Laboratory (Bar Harbor, ME). ST2 knockout C57BL/6 mice were custom-made by Cyagen (Guangzhou) Bioscience Inc. All experimental mice were maintained in micro-isolator cages under humidity free conditions in the animal facility. All experiments were performed in compliance with the guidelines of Tongji Medical College Animal Care and Use Committee.

### Heterotopic Cardiac Transplantation

Heterotopic heart transplantation was performed by a microsurgical technique. Donor hearts were transplanted into recipients through end-to-side anastomosis of the donor ascending aorta and pulmonary artery to the recipient abdominal aorta and inferior vena cava as described before (15). For cardiac transplantation models, C57BL/6 and bm12 mice were used both as donors and recipients, respectively. In single MHC-II mismatched models, bm12 mice were used as donors and B6 mice were used as recipients. Meanwhile, the *ST2^-/-^* mice received bm12 donors’ hearts as the experimental group. Donors and recipients switch roles in turning. This is an established mice cardiac transplantation model of chronic allograft rejection without immunosuppressive treatment. After cardiac transplantation, allograft impulse was assessed by daily abdominal palpation.

### General Histology and Immunohistochemical Staining

The heart allografts were resected from the recipient mouse in weeks 2, 4 and 8 weeks post transplantation. The heart specimens were fixed in 4% formaldehyde and then embedded in paraffin, sections were cut into 4μm thickness. The heart sections were stained with haematoxylin and eosin (H&E) staining to access the general histology. In addition, immunohistochemical staining was performed to determine the infiltration of inflammatory situation. In brief, the deparaffinized sections were quenched in 3% H_2_O_2_ for 30 minutes followed by antigen retrieval in sodium citrate (pH6.0) at 98°C for 20 minutes. Subsequently, sections were incubated with antibodies against CD3 (1:200, #PB9093, Boster Ltd., Wuhan, China), CD20 (1:500, #GB11540, Servicebio, Wuhan, China), and F4/80 (1:200, #70076, Cell Signaling Technology, Inc., Danvers, MA, USA) over night at 4°C to detect the infiltration of T cell, B cell and macrophage into heart, respectively. Next, 3,3’-diaminobenziding (DAB) were used as a chromogenic substrate. Statistical analysis of the histological sections was performed to quantitatively determine the amount of inflammatory cells at 2, 4, and 8 weeks. Graft rejection was graded on the extent of infiltration and the anatomical localization of inflammatory cells according to the International Society of Heart and Lung Transplantation (ISHLT) standard (16). In addition, the heart sections were also stained with Masson trichrome (#G1006, Servicebio, Wuhan, China) which differentially stains the nucleus, muscle tissue, and collagen. After H&E staining, immunohistochemical staining or Masson trichrome staining, each section was observed under light microscope at 200× and 400× magnifications, and at least 2 arteries were obtained in the same section. Quantitative analysis was performed by using the image analytical software ImageJ (Media Cybernetics, Rockville, MD, USA) to determine the histological changes after the heart transplantation for 2, 4, and 8 weeks.

### Statistical Analysis

Experimental data are expressed as the mean ± standard error of the mean (SEM). Statistical analysis was performed using Prism 6.0 (GraphPad Software, San Diego, CA) and statistical tested use indicated in the figure legends. A value of *P*<0.05 was considered statistically significant.

## Results

### Recipients ST2 Deficiency Exacerbated and Allograft ST2 Deficiency Alleviated Allograft Vasculopathy

**Figure 1:**
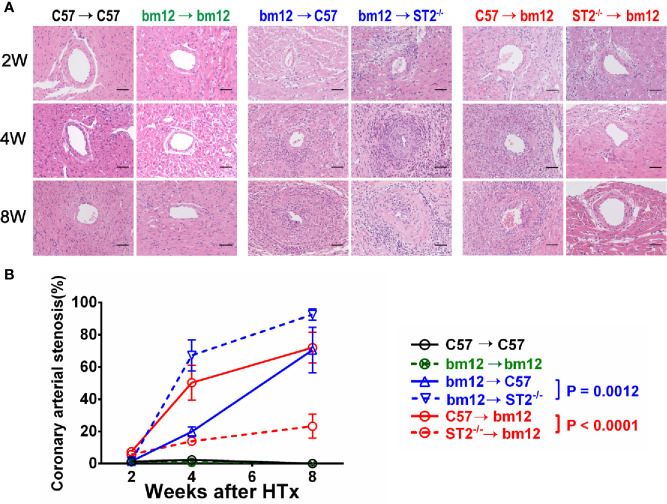
The effects of ST2 absence in cardiac graft or recipients on cardiac allograft vasculopathy. bm12 cardiac grafts were transplanted into wild type (WT) or *ST2* ^-/-^ C57 recipients. Cardiac grafts from WT or *ST2* ^-/-^ C57 mice were transplanted into bm12 recipients (n=3-6 per group). **(A)** H&E staining of allograft artery harvested on week 2,4 and 8. **(B)** Quantification of vasculopathy luminal occlusion (n = 3-6 per group) in the allografts. The percentage of vascular occlusion area was quantified by Image J. Coronary arterial stenosis (%) = (Area of internal lamina − Area of lamina)/Area of internal lamina×100% (17). Data are shown as mean ± SEM. Scale bars 50 µm. *P* values were established by 2-way ANOVA.

To ascertain the potential impact of ST2 deficiency on CAV, we used the chronic heart transplantation rejection mice model (heart transplantation in between bm12 mouse and C57 mouse). As shown in **Supplementary Figure 1A**, the pathological features of the left and right ventricle of the cardiac allograft after transplantation were different. In addition, the coronary artery is composed of intima which comprises endothelial cells, the media consisting of smooth muscle cells, and adventitia which contains fibroblasts and other cell types. In this study, we mainly observed the allograft artery in the outer tissue of left ventricle free wall **(** **Supplementary Figure 1B** **)**. In the syngeneic groups (bm12→bm12 or C57→C57) **(** **Figure 1A**, left panel), there were no intimal hyperplasia and stenosis and no inflammatory cells infiltration in the intima and adventitia of cardiac allograft arteries. The intimal cells of cardiac allograft arteries were monolayer and neatly glabrate. After bm12 hearts were transplanted into C57 recipients (bm12→C57 group), the intima of allograft artery had no significant variation and the adventitia was infiltrated a few of inflammatory cells at week 2 (**Figure 1A**, middle panel, up). At week 4, the intima of the allograft artery significantly thickened, complicating vascular stenosis and the infiltration of inflammatory cells (**Figure 1A**, middle panel, middle). At week 8, the vascular stenosis, the infiltration of inflammatory cells in the intima and adventitia, as well as the intimal hyperplasia further aggravated. The allograft artery was almost totally occluded (**Figure 1A**, middle panel, down). Compared with bm12→C57 group, ST2 deficiency in recipients (bm12→ST2^-/-^ group) markedly aggravated intimal hyperplasia, vascular stenosis and immune cells infiltration of allograft artery at both week 4 and 8 (**Figures 1A**, middle panel, **B**, blue line). The pathological features of coronary artery in the transplanted heart of group C57→bm12 were similar with that in group bm12→C57 (**Figure 1A**, right panel). There was no statistically significant difference between the two groups at 2 weeks, 4 weeks and 8 weeks (*P* value was 0.9505, 0.1449 and 0.4570). However, compared with C57→bm12 group, deficiency of ST2 in cardiac allografts (ST2^-/-^→bm12 group) markedly alleviated the coronary arterial stenosis at week 4 and 8 (**Figures 1A**, right panel, B red line). These results suggested that the ST2 signaling in the recipient prevents, and in the transplanted heart enhance, the cardiac allograft vasculopathy.

### Recipients ST2 Deficiency Exacerbated and Allograft ST2 Deficiency Alleviated Allograft Vasculopathy Fibrosis

**Figure 2:**
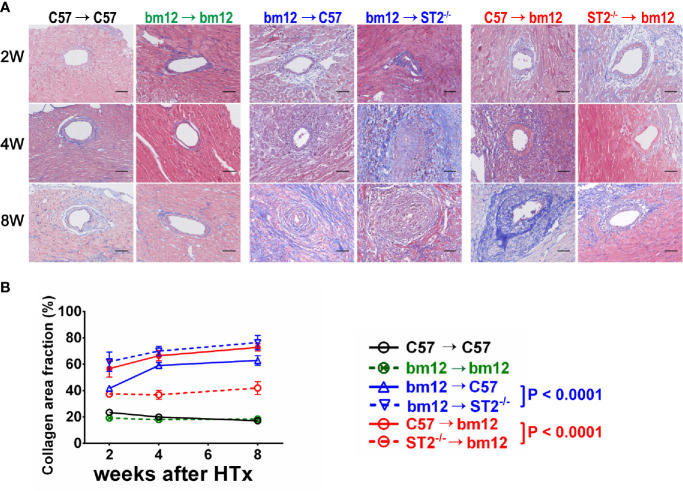
The effects of recipient or allograft ST2 deficiency on the fibrosis of vasculopathy. **(A)** Masson staining of allografts artery harvested on week 2, 4 and 8. **(B)** Quantification of collagen area fraction in vasculopathy (n = 3-6 per group) using Image (J) The cardiac allograft vasculopathy collagen area fraction (%) = Area of artery collagen volume/Area of artery cross sections×100%. Data are shown as mean ± SEM. Scale bars are 50 µm. *P* values were established by 2-way ANOVA.

To further confirm the effects of ST2 deficiency on CAV, the quantitative Masson staining was used to detect the vasculopathy fibrosis. In the syngeneic groups, the fibrosis of the allograft artery was not obvious **(** **Figure 2A**, left panel). In bm12→C57 group, the fibrosis of the allograft artery gradually increased and arrived the peak at week 8 **(** **Figure 2A**, left panel). Compared with bm12→C57 group, ST2 deficiency in recipients (bm12→ST2^-/-^ group) markedly aggravated the fibrosis of the allograft artery, especially at week 4 and 8. **(** **Figures 2A**, middle panel, **B**). The fibrosis of allograft artery C57→bm12 group were similar with that in group bm12→C57 at each time point **(** **Figure 2A**, right panel). There was no statistically significant difference between the two groups at 2 weeks, 4 weeks and 8 weeks (*P* value was 0.0832, 0.1779 and 0.0958). ST2 deficiency in cardiac allograft (ST2^-/-^→bm12) decreased the fibrosis of allograft artery significantly **(** **Figures 2A**, right panel, **B**). Next, we further observed the characteristics of the fibrosis of vasculopathy in detail. As illustrated in **Supplementary Figure 2A**, in bm12→C57 group, the fibrosis of allograft arteries major existed in adventitia at week 2. Accompanying the development of CAV, the fibrosis in intima increased gradually. Recipients ST2 deficiency significantly enhanced the percentage of intima fibrosis in the total fibrosis of vasculopathy, while an opposite tendency was displayed when allograft ST2 was deleted. These results suggested that recipient-derived ST2 signaling inhibited and graft-derived ST2 signaling accelerated the fibrosis of vasculopathy.

### Recipients ST2 Deficiency Increased and Allograft ST2 Deficiency Decreased Macrophage Infiltration in Allografts

**Figure 3:**
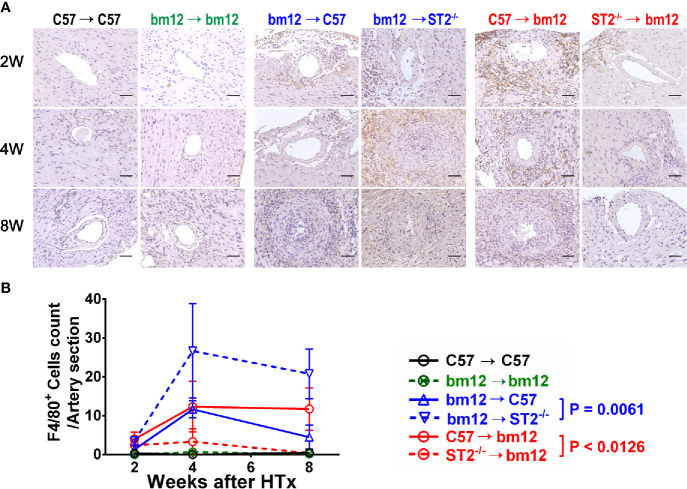
The effects of recipient or graft ST2 deficiency on F4/80^+^ macrophages infiltration in cardiac allograft. **(A)** The allograft infiltrated F4/80^+^ macrophages identified using IHC. **(B)** Quantification of infiltrated F4/80^+^ macrophages (n = 4-6 per group) in the arterial wall of allografts with Image (J) HTx: heart transplantation. Data are presented as mean ± SEM. Scale bars are 50 µm. *P* values were established by 2-way ANOVA.

To investigate whether the immune cells infiltration was associated with the influence of ST2 deficiency on cardiac allograft vasculopathy, the impact of ST2 deficiency on macrophage infiltration was detected *via* immunohistochemistry. As shown in **Figure 3**, there were no obvious macrophage (F4/80^+^ cells) infiltration in the arterial wall of allograft in syngeneic groups **(** **Figures 3A**, left panel, **B**, black line and green line). In the control group (bm12→C57), there was a few of F4/80^+^ macrophages infiltration in the arterial wall of the allograft at week 2 **(** **Figure 3A**, middle panel). Subsequently, at week 4 and 8, the infiltrated macrophages increased. Deficiency of ST2 in recipients markedly increased F4/80^+^ macrophages infiltration to the maximum at week 4 **(** **Figures 3A**, middle panel, **B**, blue lines). In C57→bm12 group, there was the maximum infiltration of F4/80^+^ macrophages in the arterial wall at week 4 **(** **Figures 3A**, right panel, **B**). The F4/80^+^ macrophages infiltration in the arterial wall of the allograft C57→bm12 group were similar with that in group bm12→C57 at each time point **(** **Figures 3A**, right panel, **B**). There was no statistically significant difference between the two groups at 2 weeks, 4 weeks and 8 weeks (*P* value was 0.2260, 0.9271 and 0.2906). However, at week 8, the number of infiltrated macrophages no obvious changed compared with that at week 4 **(** **Figures 3A**, right panel, **B**, red solid line). ST2 deficiency in cardiac allograft markedly diminished the infiltration F4/80^+^ macrophages in the coronary arterial wall at week 2, 4 and 8 **(** **Figures 3A**, right panel, **B**, red dotted line).

Next, we further observed the macrophages infiltration in the arterial intima and adventitia respectively. Recipients ST2 deficiency increased the macrophages infiltration either in the arterial intima or adventitia **(** **Supplementary Figures 3A, B** **)**. Allograft ST2 deficiency decreased macrophages infiltration in both arterial intima and adventitia **(** **Supplementary Figures 3C, D** **)**. These results suggested that the CAV and fibrosis degree alteration induced by ST2 deficiency is positively associated with the number of infiltrated macrophages.

### Recipients ST2 Deficiency Increased and Allograft ST2 Deficiency Decreased CD3^+^ T Cells Infiltration in Allografts

**Figure 4:**
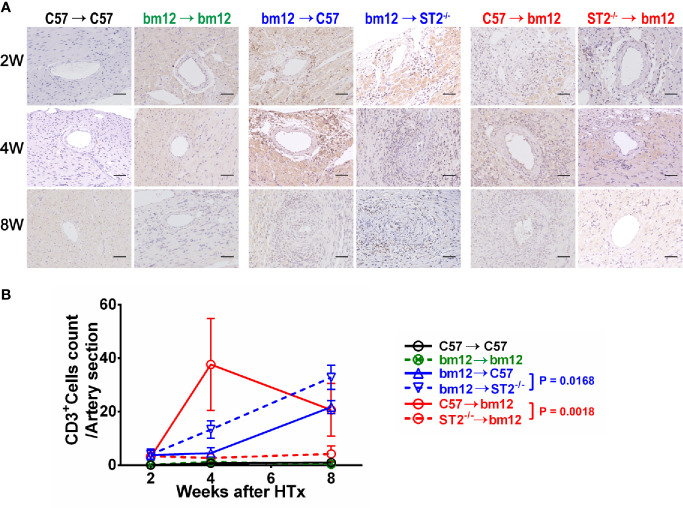
The effects of recipient or graft ST2 deficiency on CD3^+^ T cells infiltration in cardiac allograft. **(A)** The allograft infiltrated CD3^+^ T cells identified using IHC. **(B)** Quantification of infiltrated CD3^+^ T cells (n = 4-6 per group) in the arterial wall of allografts with Image J. HTx: heart transplantation. Data are presented as mean ± SEM. Scale bars are 50 µm. *P* values were established by 2-way ANOVA.

To confirm whether the specific immune cells infiltration was associated with the influence of ST2 deficiency on cardiac allograft vasculopathy, the impact of ST2 deficiency on T cells infiltration was detected *via* immunohistochemistry. As shown in **Figure 4**, there were no obvious T cells (CD3^+^ cells) infiltration in the arterial wall of allograft in syngeneic groups (**Figures 4A**, left panel, **B**, black line and green line). In the control group (bm12→C57), there was a few of CD3^+^ T cells infiltration in the arterial wall of the graft at week 2 **(** **Figure 4A**, middle panel). Subsequently, at week 4 and 8, the infiltrated CD3^+^ T cells increased. Deficiency of ST2 in recipients markedly increased CD3^+^ T cells infiltration to the maximum at week 8 **(** **Figures 4A**, middle panel, **B**, blue lines). In C57→bm12 group, there was the maximum infiltration of CD3^+^ T cells in the arterial wall at week 4 **(** **Figures 4A**, right panel, **B**). The CD3^+^ T cells infiltration in the arterial wall of the allograft C57→bm12 group were similar with that in group bm12→C57 at each time point **(** **Figures 4A**, right panel, **B**). There was no statistically significant difference between the two groups at 2 weeks, 4 weeks and 8 weeks (*P* value was 0.7389, 0.0724 and 0.8444). However, at week 8, the number of infiltrated CD3^+^ T cells significantly decreased compared with that at week 4 **(** **Figures 4A**, right panel, **B** red solid line). ST2 deficiency in cardiac allograft markedly diminished the infiltration CD3^+^ T cells in the coronary arterial wall at week 2, 4 and 8 **(** **Figures 4A**, right panel, **B**, red dotted line).

Next, we further observed the CD3^+^ T cells infiltration in the arterial intima and adventitia respectively. Recipients ST2 deficiency increased the CD3^+^ T cells infiltration either in the arterial intimae or adventitia **(** **Supplementary Figures 4A, B** **)**. Allograft ST2 deficiency decreased CD3^+^ T cells infiltration in both arterial intimae and adventitia **(** **Supplementary Figures 4C, D** **)**. These results suggested that the CAV and fibrosis degree alteration induced by ST2 deficiency is positively associated with the number of infiltrated CD3^+^ T cells.

### Recipients ST2 Deficiency Had No Impact on and Allograft ST2 Deficiency Decreased CD20^+^ B Cells Infiltration in Allografts

**Figure 5:**
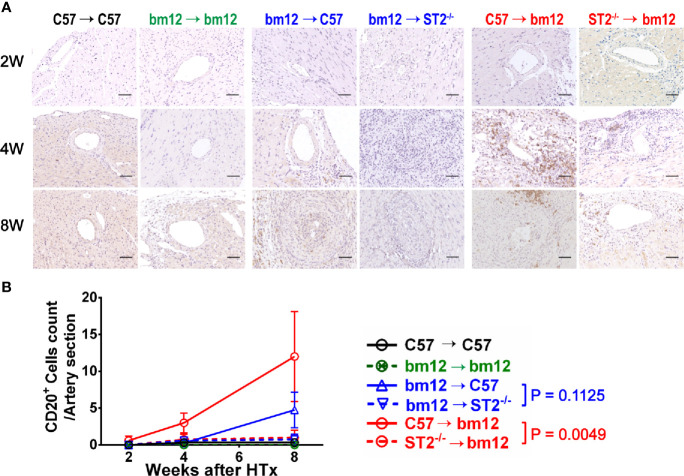
The effects of recipient or graft ST2 deficiency on CD20^+^ B cells infiltration in cardiac allograft. **(A)** The allograft infiltrated CD20^+^ B cells identified using IHC and quantified with Image J. **(B)** Quantification of vasculopathy infiltrated CD20^+^ B cells (n = 4-6 per group) in the wall of the allograft artery. Data are shown as mean ± SEM. Scale bars are 50 µm. *P* values were established by 2-way ANOVA.

We further investigated whether the B cells infiltration was associated with the influence of ST2 deficiency on cardiac allograft vasculopathy. As shown in **Figure 5**, there were no obvious B cells (CD20^+^ B cells) infiltration in the arterial wall of allograft in syngeneic groups **(** **Figures 5A**, left panel, **B**, black line and green line). In the control group (bm12→C57), there was few of CD20^+^ B cells infiltration in the arterial wall of the graft at the week 2 **(** **Figure 5A**, middle panel). Subsequently, at week 4 and 8, the infiltrated CD20^+^ B cells slightly increased. Deficiency of ST2 in recipients slightly mitigated CD20^+^ B cells infiltration at week 4 and 8, but no statistical significance **(** **Figures 5A**, middle panel, **B**, blue lines). In C57→bm12 group, the number of infiltrated CD20^+^ B cells in the arterial wall gradually increased to the maximum at week 8. The CD20^+^ T cells infiltration in the arterial wall of the allograft C57→bm12 group were similar with that in group bm12→C57 at each time point **(** **Figures 5A**, right panel, **B**). There was no statistically significant difference between the two groups at 2 weeks, 4 weeks and 8 weeks (*P* value was 0.3466, 0.1167 and 0.2712). ST2 deficiency in cardiac allograft markedly diminished the infiltration of CD20^+^ B cells in the arterial wall at week 4 and 8 (**Figures 5A**, right panel, **B**, red lines).

Next, we further observed the CD20^+^ B cells infiltration in the arterial intimae and adventitia respectively. Recipients ST2 deficiency significantly influence the CD20^+^ B cells infiltration neither in the arterial intima nor in the adventitia **(** **Supplementary Figures 5A, B** **)**. Allograft ST2 deficiency decreased CD20^+^ B cells infiltration in both arterial intimae and adventitia **(** **Supplementary Figures 5C, D** **)**. These results suggested that the CAV and fibrosis degree alteration induced by allograft ST2 deficiency is positively associated with the number of infiltrated CD20^+^ B cells.

## Discussion

While great improvement has been achieved in the precaution of acute rejection, CAV is still primarily resistant to therapy. The immunopathology of CAV is not well understood (18). The aim of this study was to accurately address the role of IL-33/ST2 signaling in cardiac allograft vasculopathy *via* deleting ST2 in allograft or recipient respectively. The ST2 deficiency of the recipient mainly reflects the effects of IL-33/ST2 signaling on immune cells, and the ST2 deficiency of the donor reflects the effects on allograft resident cells. This research method is more conducive to analyze the precise role of IL-33/ST2 signaling in the allograft and recipient. In clinical practice, although the immunosuppressive drugs effectively inhibit immune rejection, they have several side effects and increase the risk of infection. Therefore, it is another choice to intervene regional immunity happened in allograft with relatively few side effects and better curative outcome. Our results suggest that the allograft IL-33/ST2 signal plays obvious roles in the development of CAV, and intervention of the allograft IL-33/ST2 signal may have a better effect. We further observed the local characteristics of CAV. The pathological characteristics of the graft artery were analyzed by dividing it into intima and media. The aim is to more accurately describe the pathological characteristics of each layer of allograft artery tissue and to provide a precise therapeutic target. The tissue sections selected for immunohistochemistry were continuous and similar in structure, which could reflect the spatial location of infiltrated immune cells in CAV and supply the clues communication between different subsets. Immunohistochemistry was an important method to explore the details of the development of CAV.

Our results demonstrate that recipients ST2 deficiency exacerbated fibrosis of vasculopathy. The fibrosis is the major characteristic of the CAV development. In the process of chronic rejection after heart transplantation, specific immune cells infiltrated into the heart graft vessels *via* blood or lymph circulation. Recent studies addressed that ST2 is expressed on many immune cells, including macrophages, and T cells, in particular Th2 cells (10). Our data revealed that the local vascular fibrosis in the allografts of ST2-deficiency recipients accompanied with immune cells, and clear majority of infiltrated immune cells were CD3^+^ T cells and F4/80^+^ macrophages. The enhanced local inflammatory response may be related to the increased collagen deposition. Consequently, it can be inferred that the IL-33/ST2 signal of the recipient may influence local fibrosis by impacting the inflammatory cells infiltration. By contrast, allograft ST2 deficiency alleviated allograft vasculopathy fibrosis, especially the intima. It may be related to the decrease of IL-33/ST2 signal-related inflammatory chemokines and pro-inflammatory cytokine that determine the immune cells infiltration in the allograft artery. Li et al. reported that the absence of graft IL-33 resulted in increased chronic allograft vasculopathy and fibrosis (12) which was consistent with our results. In our study, the expression of IL-33 in the heart grafts were not affected in the ST2 knockout donors, and the expression of ST2 in immune cells from the recipients were normal as well. Consequently, the IL-33/ST2 signal could play a protective role normally. However, IL-33 effects, although mostly cardioprotective, vary depending on the disease state and cell type. ST2L is constitutively expressed on cells of the cardiovascular system, in particular endothelial cells (19). Several studies have reported direct activation and pro-inflammatory effects of IL-33 on endothelial cells which is the main cause of CAV (20, 21). Therefore, it can be speculated that the activation endothelial cells were reduced when ST2 deficiency in the transplanted heart, and then the CAV was relieved. This is also the focus of the mechanism we want to explore next. In the same way, it can be inferred that in ST2 knockout recipient mice, the expression of ST2 in the heart grafts endothelial cells were normal, there may be some factors that can promote the development of CAV. In addition, the deficiency of ST2 in the recipient may affect the number and function of immune cells infiltrating the transplanted heart. And this speculation needs further verification. In summary, Li’s results are similar to us in exploring the role of IL-33/ST2 signaling in CAV. Besides, our study focused on the role of ST2 in cardiac resident cells and immune cells, which could further explain the mechanism during the development of CAV.

In a word, we provide the evidences that the bioactivities of IL-33/ST2 signaling in CAV are pleiotropy. IL-33/ST2 signaling may inhibit the development of CAV *via* acting on immune cells and facilitate the CAV *via* acting on allograft resident cells. The clinical significance of these findings is that blockade of IL-33/ST2 signal in cardiac allograft may offer new strategy to delay the development of CAV.

## Data Availability Statement

The original contributions presented in the study are included in the article/**Supplementary Material**. Further inquiries can be directed to the corresponding author.

## Ethics Statement

The animal study was reviewed and approved by Tongji Medical College Animal Care and Use Committee.

## Author Contributions

FZ worked on conception and design. Acquisition of data and analysis was conducted by ZZ and NZ. ZZ performed the majority of the experiments, and JS, CD, SW, MJ, XT, YL, XL, YX, ZT, and FG contributed to the experimentation. All authors contributed to the article and approved the submitted version.

## Funding

This work was supported by the National Natural Science Foundation of China (Grant No. 31670876) and (Grant No. 31470852).

## Conflict of Interest

The authors declare that the research was conducted in the absence of any commercial or financial relationships that could be construed as a potential conflict of interest.

## Supplementary Material

Supplementary Figure 1 The pathological features of cardiac allograft in chronic transplantation rejection mice model. C57BL/6 cardiac allografts were transplanted into bm12 recipients (n=3 per group). **(A)** Grafts harvested at week 4 were assessed by H&E staining and percentage of vascular occlusion area was quantified by Image J. Quantification of vasculopathy luminal area (n=3 per group) in the allografts. Abbreviations used in this figure: RV: right ventricular; LV: left ventricular; I, Intima; M, Media; A, Adventitia. **(B)** The representative allograft coronary artery section image post transplantation 4 weeks. The dotted area represents the part we sampled arteries which diameters were greater than 50μm. Data were shown as mean ± SEM. Scale bars= 50µm. *P* values established by unpaired Student’s t-test. ***P*<0.01.

Supplementary Figure 2 The Masson staining of coronary artery in cardiac allograft. **(A)** bm12 grafts were transplanted into C57BL/6 or ST2-deficient (*ST2* ^-/-^) C57BL/6 recipients (n≥3 per group). The graft collagen volume fraction identified using Masson staining (n = 4-6 per group) in the allografts. The green dotted line represents the elastic layer. The black dotted line indicates the media layer. The red dotted bordered stands for artery lumen. The thickening intima of artery is between the red and green dotted line. Between the green dotted line and the black dotted line are the medial and media parts of the artery. **(B)** Quantification of vasculopathy collagen volume fraction (n = 4-6 per group) in the allografts. **(C)** C57BL/6 or ST2-deficient (*ST2* ^-/-^) C57BL/6 grafts were transplanted into bm12 recipients (n ≥ 3 per group). **(D)** Quantification of vasculopathy collagen volume fraction (n = 4-6 per group) in the allografts. In the diagram, the red dotted line indicates the intima layer. Data were shown as mean ± SEM. Scale bars = 50µm. *P* values were established by 2-way ANOVA.

Supplementary Figure 3 F4/80^+^ macrophages infiltration in allografts in ST2 deficiency recipients or just graft ST2 deficiency recipients. **(A, C)** The graft infiltrating F4/80^+^ macrophages identified using IHC and quantified with Image J. In the diagram, the red dotted line indicates the intima layer. The green dotted line represents the elastic layer. The black dotted line indicates the media layer. The red dotted bordered stands for artery lumen. The thickening intima of artery is between the red and green dotted line. Between the green dotted line and the black dotted line are the medial and media parts of the artery. **(B, D)** Quantification of vasculopathy infiltrating F4/80^+^ macrophages (n = 4-6 per group) in the allografts. Data were shown as mean ± SEM. Scale bars 50µm. *P* values were established by 2-way ANOVA.

Supplementary Figure 4 The effects of recipient or graft ST2 deficiency on CD3^+^ T cells infiltration in cardiac allograft. **(A, C)** The graft infiltrated CD3^+^ T cells were identified using IHC and quantified with Image J. The red dotted circle indicates the intimae layer and bordered artery lumen. The green dotted circle represents the elastic layer. The black dotted circle indicates the media layer. The thickening intimae of artery is between the red and green dotted circle. Between the green dotted circle and the black dotted circle is the medial layer of the artery. **(B, D)** Quantification of vasculopathy infiltrating CD3^+^ cells (n = 4-6 per group) in the allografts. Data were shown as mean ± SEM. Scale bars = 50µm. *P* values were established by 2-way ANOVA.

Supplementary Figure 5 The effect of recipient or graft ST2 deficiency on CD20^+^ B cells infiltration in allograft artery. **(A, C)** The infiltrated CD20^+^ B cells were identified using IHC and quantified with Image J. In A and C, the red dotted circle indicates the intimae layer. The green dotted circle represents the elastic layer. The black dotted circle indicates the media layer. The red dotted circle bordered artery lumen. The thickening intimae of artery is between the red and green dotted circle. Between the green dotted circle and the black dotted circle, it is the medial layer of the artery. **(B, D)** Quantification of vasculopathy infiltrating CD20^+^ cells (n = 4-6 per group) in the allografts. Data were shown as mean ± SEM. Scale bars = 50µm. n = 4-6 per group. *P* values were established by 2-way ANOVA.
