# Supplementary figures and images for: Allograft or Recipient ST2 Deficiency Oppositely Affected Cardiac Allograft Vasculopathy via Differentially Altering Immune Cells Infiltration
Source: Front Immunol. 2021 Mar 18;12:657803. doi: 10.3389/fimmu.2021.657803 (PMC8012811; doi:10.3389/fimmu.2021.657803)

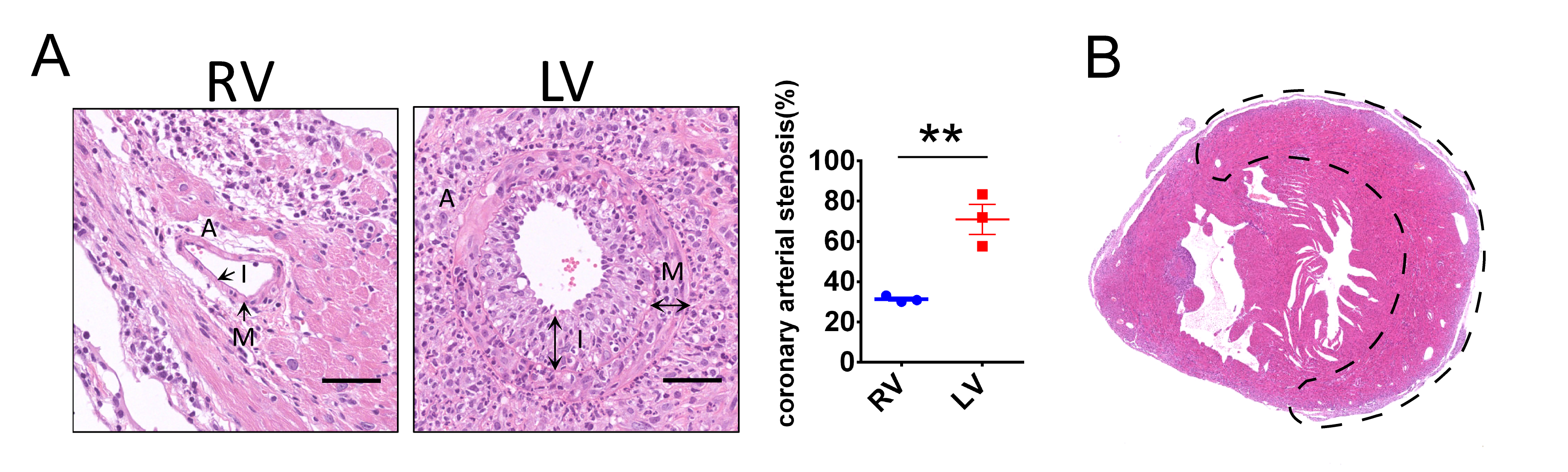

Supplement: Supplementary Figure 1 — The pathological features of cardiac allograft in chronic transplantation rejection mice model. C57BL/6 cardiac allografts were transplanted into bm12 recipients (n=3 per group). (A) Grafts harvested at week 4 were assessed by H&E staining and percentage of vascular occlusion area was quantified by Image J. Quantification of vasculopathy luminal area (n=3 per group) in the allografts. Abbreviations used in this figure: RV: right ventricular; LV: left ventricular; I, Intima; M, Media; A, Adventitia. (B) The representative allograft coronary artery section image post transplantation 4 weeks. The dotted area represents the part we sampled arteries which diameters were greater than 50μm. Data were shown as mean ± SEM. Scale bars= 50µm. P values established by unpaired Student’s t-test. **P<0.01. [file Image_1.tif]

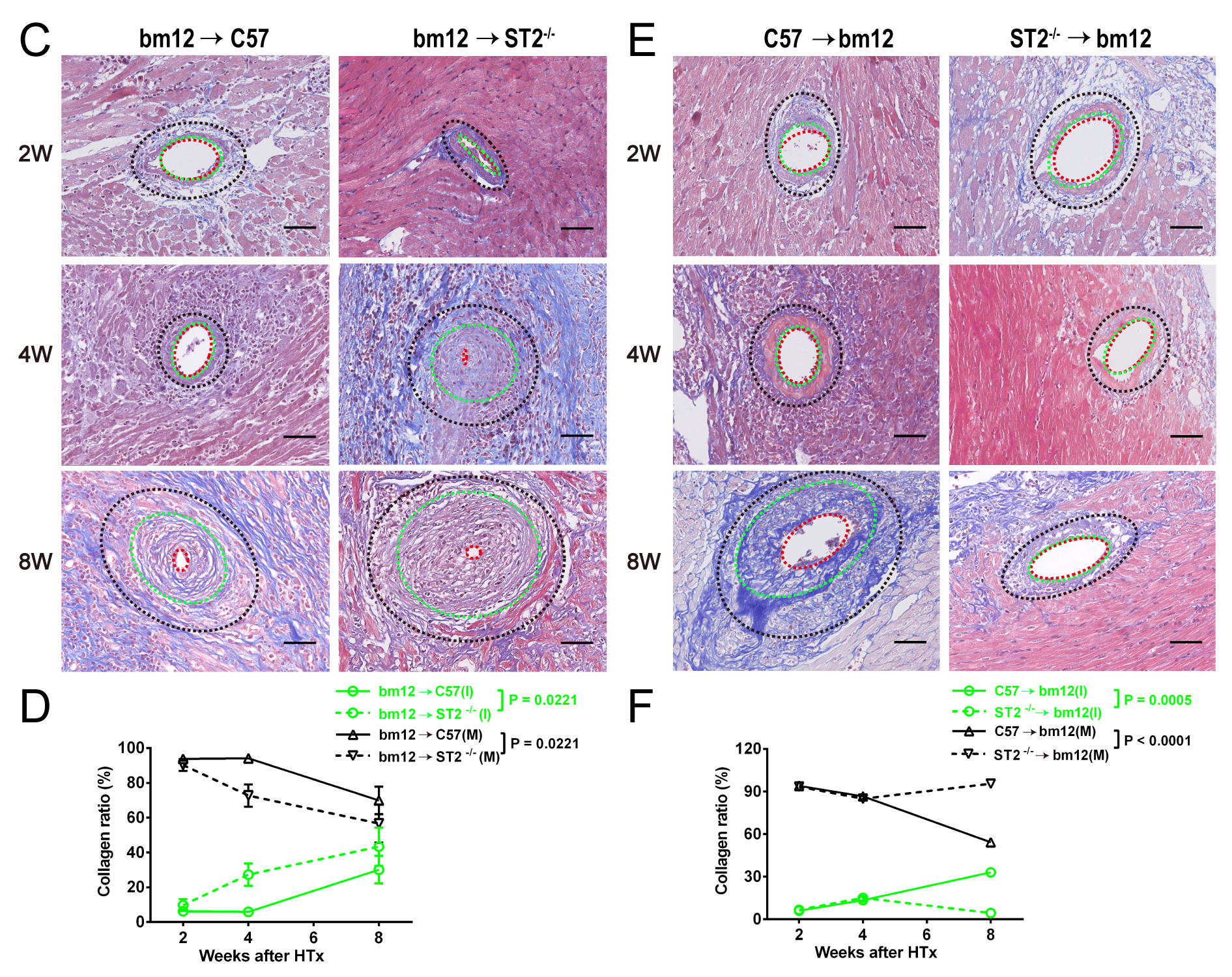

Supplement: Supplementary Figure 2 — The Masson staining of coronary artery in cardiac allograft. (A) bm12 grafts were transplanted into C57BL/6 or ST2-deficient (ST2 -/-) C57BL/6 recipients (n≥3 per group). The graft collagen volume fraction identified using Masson staining (n = 4-6 per group) in the allografts. The green dotted line represents the elastic layer. The black dotted line indicates the media layer. The red dotted bordered stands for artery lumen. The thickening intima of artery is between the red and green dotted line. Between the green dotted line and the black dotted line are the medial and media parts of the artery. (B) Quantification of vasculopathy collagen volume fraction (n = 4-6 per group) in the allografts. (C) C57BL/6 or ST2-deficient (ST2 -/-) C57BL/6 grafts were transplanted into bm12 recipients (n ≥ 3 per group). (D) Quantification of vasculopathy collagen volume fraction (n = 4-6 per group) in the allografts. In the diagram, the red dotted line indicates the intima layer. Data were shown as mean ± SEM. Scale bars = 50µm. P values were established by 2-way ANOVA. [file Image_2.tif]

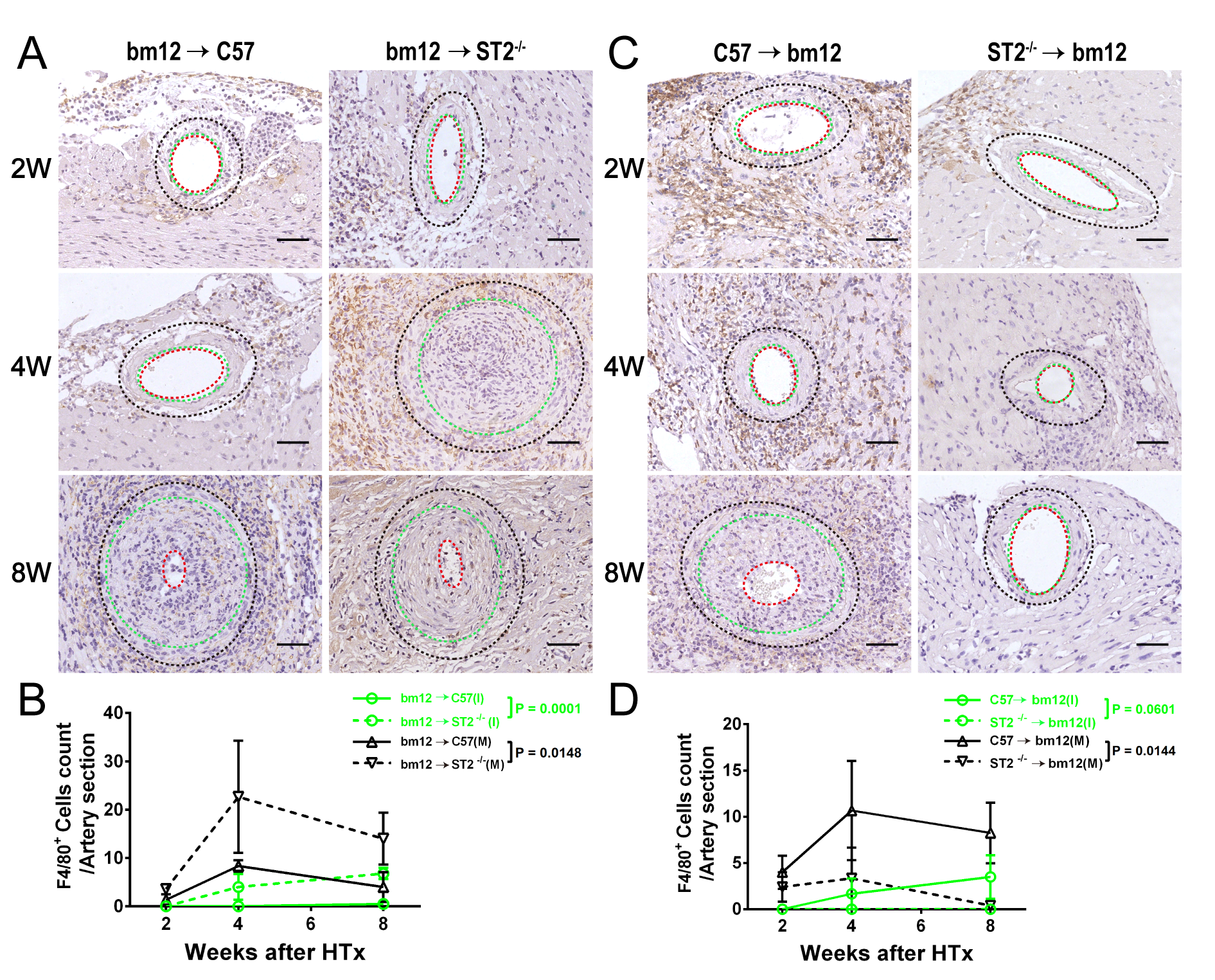

Supplement: Supplementary Figure 3 — F4/80+ macrophages infiltration in allografts in ST2 deficiency recipients or just graft ST2 deficiency recipients. (A, C) The graft infiltrating F4/80+ macrophages identified using IHC and quantified with Image J. In the diagram, the red dotted line indicates the intima layer. The green dotted line represents the elastic layer. The black dotted line indicates the media layer. The red dotted bordered stands for artery lumen. The thickening intima of artery is between the red and green dotted line. Between the green dotted line and the black dotted line are the medial and media parts of the artery. (B, D) Quantification of vasculopathy infiltrating F4/80+ macrophages (n = 4-6 per group) in the allografts. Data were shown as mean ± SEM. Scale bars 50µm. P values were established by 2-way ANOVA. [file Image_3.tif]

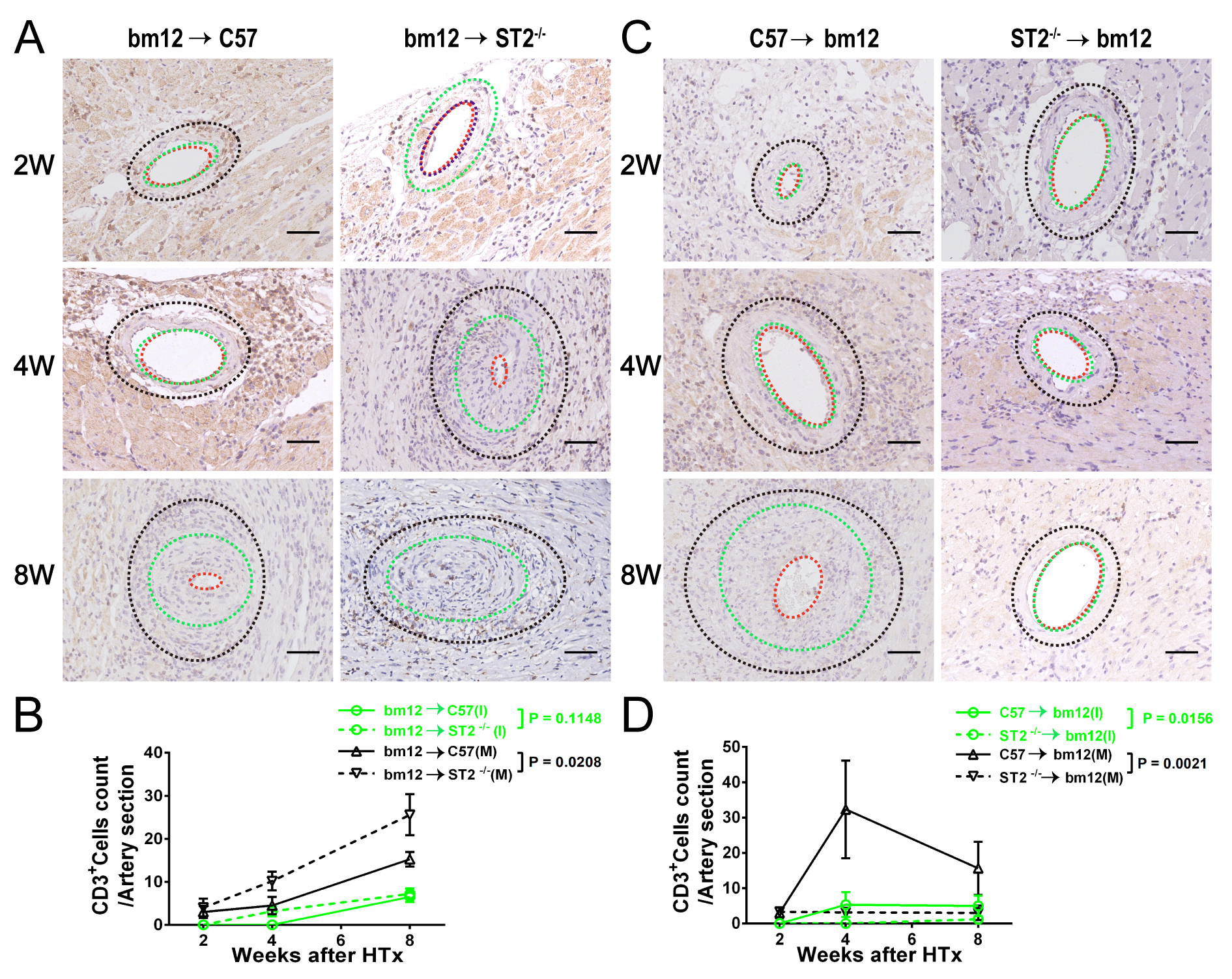

Supplement: Supplementary Figure 4 — The effects of recipient or graft ST2 deficiency on CD3+ T cells infiltration in cardiac allograft. (A, C) The graft infiltrated CD3+ T cells were identified using IHC and quantified with Image J. The red dotted circle indicates the intimae layer and bordered artery lumen. The green dotted circle represents the elastic layer. The black dotted circle indicates the media layer. The thickening intimae of artery is between the red and green dotted circle. Between the green dotted circle and the black dotted circle is the medial layer of the artery. (B, D) Quantification of vasculopathy infiltrating CD3+ cells (n = 4-6 per group) in the allografts. Data were shown as mean ± SEM. Scale bars = 50µm. P values were established by 2-way ANOVA. [file Image_4.tif]

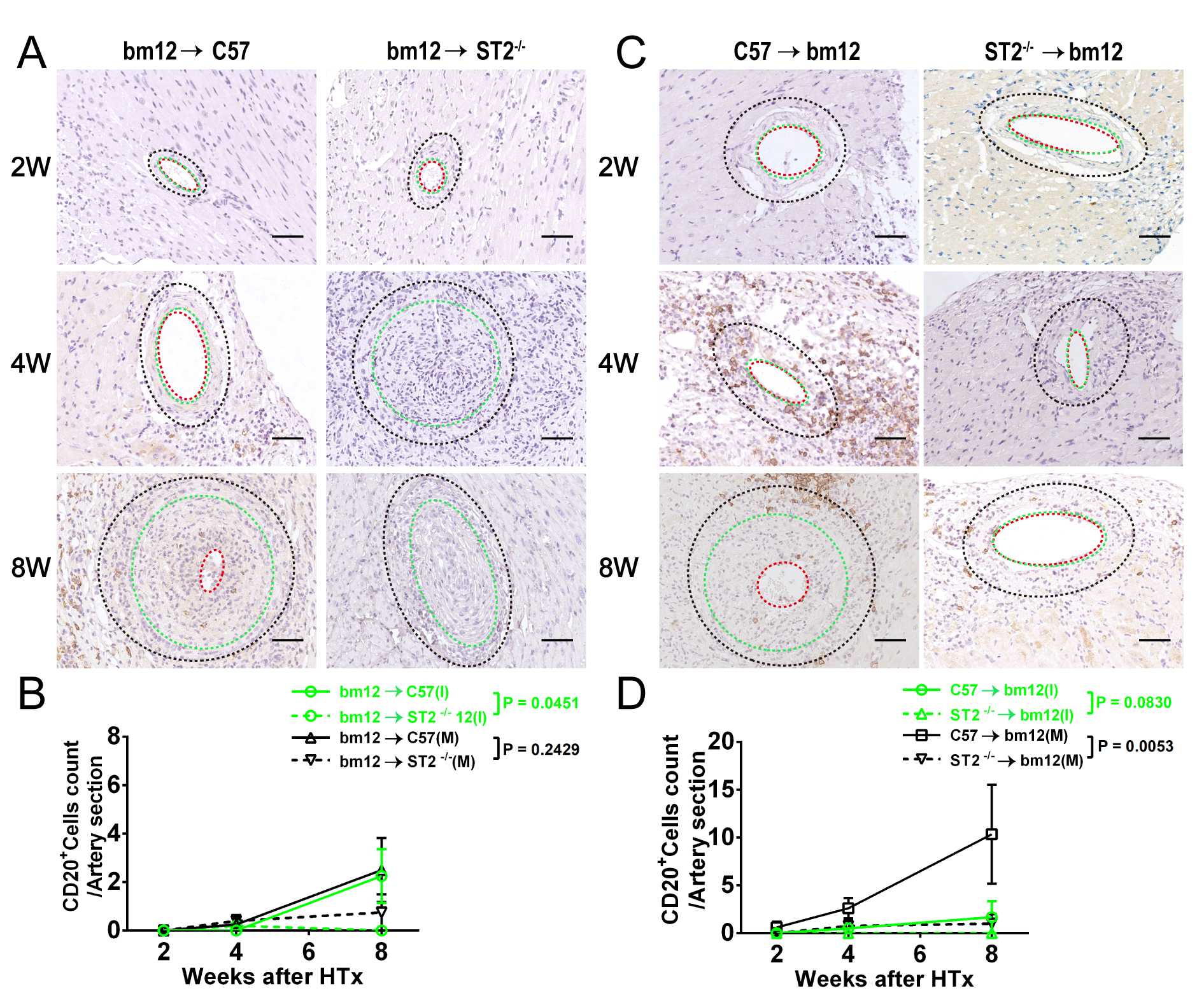

Supplement: Supplementary Figure 5 — The effect of recipient or graft ST2 deficiency on CD20+ B cells infiltration in allograft artery. (A, C) The infiltrated CD20+ B cells were identified using IHC and quantified with Image J. In A and C, the red dotted circle indicates the intimae layer. The green dotted circle represents the elastic layer. The black dotted circle indicates the media layer. The red dotted circle bordered artery lumen. The thickening intimae of artery is between the red and green dotted circle. Between the green dotted circle and the black dotted circle, it is the medial layer of the artery. (B, D) Quantification of vasculopathy infiltrating CD20+ cells (n = 4-6 per group) in the allografts. Data were shown as mean ± SEM. Scale bars = 50µm. n = 4-6 per group. P values were established by 2-way ANOVA. [file Image_5.tif]
